# A tale of two lineages: how the strains of the earliest divergent symbiotic *Frankia* clade spread over the world

**DOI:** 10.1186/s12864-022-08838-5

**Published:** 2022-08-19

**Authors:** Fede Berckx, Thanh Van Nguyen, Cyndi Mae Bandong, Hsiao-Han Lin, Takashi Yamanaka, Sae Katayama, Daniel Wibberg, Jochen Blom, Jörn Kalinowski, Masaki Tateno, Jessica Simbahan, Chi-Te Liu, Andreas Brachmann, Katharina Pawlowski

**Affiliations:** 1 Department of Ecology, Environment and Plant Sciences, Stockholm University, 106 91 Stockholm, Sweden; 2 College of Science, University of the Philippines, Diliman, Quezon City, Philippines; 3 Institute of Biotechnology, National Taiwan University, Taipei, 10617 Taiwan; 4 Institute of Plant and Microbial Biology, Academia Sinica, Taipei, 11529 Taiwan; 5 Forest Research and Management Organization, Nabeyashiki, Morioka, Iwate, 020-0123 Japan; 6 Nikko Botanical Garden, Department of Biological Sciences, Graduate School of Science, The University of Tokyo, Tochigi, 321-1435 Japan; 7 Center for Biotechnology (CeBiTec), Bielefeld University, 33594 Bielefeld, Germany; 8 Bioinformatics and Systems Biology, Justus Liebig University, 35392 Gießen, Germany; 9 Agricultural Biotechnology Research Center, Academia Sinica, Taipei, 11529 Taiwan; 10 Biocenter, LMU München, 82152 Planegg, Martinsried Germany

**Keywords:** Root nodules, *Frankia*, *Coriaria*, Actinorhizal symbiosis, Biogeography

## Abstract

It is currently assumed that around 100 million years ago, the common ancestor to the Fabales, Fagales, Rosales and Cucurbitales in Gondwana, developed a root nodule symbiosis with a nitrogen-fixing bacterium. The symbiotic trait evolved first in *Frankia* cluster-2; thus, strains belonging to this cluster are the best extant representatives of this original symbiont. Most cluster-2 strains could not be cultured to date, except for *Frankia coriariae,* and therefore many aspects of the symbiosis are still elusive. Based on phylogenetics of cluster-2 metagenome-assembled genomes (MAGs), it has been shown that the genomes of strains originating in Eurasia are highly conserved. These MAGs are more closely related to *Frankia* cluster-2 in North America than to the single genome available thus far from the southern hemisphere, i.e., from Papua New Guinea.

To unravel more biodiversity within *Frankia* cluster-2 and predict routes of dispersal from Gondwana, we sequenced and analysed the MAGs of *Frankia* cluster-2 from *Coriaria japonica* and *Coriaria intermedia* growing in Japan, Taiwan and the Philippines. Phylogenetic analyses indicate there is a clear split within *Frankia* cluster-2, separating a continental from an island lineage. Presumably, these lineages already diverged in Gondwana.

Based on fossil data on the host plants, we propose that these two lineages dispersed via at least two routes. While the continental lineage reached Eurasia together with their host plants via the Indian subcontinent, the island lineage spread towards Japan with an unknown host plant.

**Supplementary Information:**

The online version contains supplementary material available at 10.1186/s12864-022-08838-5.

## Background

The nitrogen-fixing clade encompasses all symbiotic plants able to form root nodules which host diazotrophic bacteria. These plants can be traced back to the common ancestor of the Fabales, Fagales, Cucurbitales, and Rosales. While legumes (Fabaceae, Fabales) and *Paraspona* (Cannabaceae, Rosales) engage with Gram-negative rhizobia, the remaining root nodule-forming plants all engage with Gram-positive *Frankia.* The analysis of the evolution of this symbiosis involved several different scenarios [1–3], but the most recent phylogenomic studies have shown that in the most parsimonious hypothesis the common ancestor was symbiotic and the symbiotic capability was subsequently lost in the majority of lineages [4, 5].

A detailed study on the distribution of the genus *Nothofagus* (Fagales) concluded that the order evolved in the supercontinent Gondwana [6]. The same origin was found for the genus *Coriaria* (Cucurbitales) [7]. Therefore, the common symbiotic ancestor of the nitrogen-fixing plant clade must have evolved in Gondwana. This ancestor should have had either a rhizobial or a *Frankia* microsymbiont. Van Velzen et al. [8] argued persuasively that, given the polyphyletic origin of the oxygen protection system for bacterial nitrogenase in nodules, which is required for rhizobial but not *Frankia* nitrogen fixation, the original microsymbiont cannot have been a rhizobial strain, i.e. it must have been a *Frankia* strain.

Actinobacteria from the genus *Frankia* are the microsymbionts of actinorhizal plants. Phylogenetically, *Frankia* strains can be grouped into four phylogenetic clades called clusters, three of which encompass symbiotic strains and roughly represent host specificity groups [9]. Phylogenetic analysis shows that after the split of symbiotic cluster-2 and non-symbiotic cluster-4, the precursor of the symbiotic clusters 1 and 3 split off from cluster-4 [10–14]. *Frankia* cluster-2, where the symbiotic trait evolved for the first time, is therefore of particular interest for understanding the evolution of actinorhizal symbiosis as its strains seem to represent the closest approximation of the original symbiont. The evolution of cluster-2 should yield insight into the evolution of root nodule symbiosis in general.

*Frankia* strains grow as a mycelium *ex planta*. Cluster-2 strains show low saprotrophic potential [12, 15] and were, in spite numerous unsuccessful isolation efforts, considered uncultivable until Gtari et al. [11] and Gueddou et al. [16] published the isolation of two closely related alkaliphilic strains of *Frankia coriariae,* from nodules of the Mediterranean species *Coriaria myrtifolia* that fulfilled Koch’s postulate. Generally, cluster-2 inocula represent assemblages of different strains, and not all of these strains can enter an effective symbiosis with the host plant the inoculum came from [14]. This has been illustrated by the fact that metagenome-assembled genomes (MAGs) [17] differ when sequencing whole nodules, as compared to when isolating *Frankia* vesicles – i.e. nitrogen-fixing symbiotic structures – before sequencing [14]. Some strains can enter the nodule but cannot fix nitrogen there.

*Frankia c*luster-2 strains have a wide host range and the geographic distribution of their host plants is disjunct [18]. Actinorhizal members of the order Rosales nodulated by cluster-2 strains, namely Dryadoideae (Rosaceae) and *Ceanothus* sp. (Rhamnaceae), are restricted to North America. The family Datiscaceae (Cucurbitales) consists of two species: *Datisca cannabina* which is mostly restricted to northern India/Pakistan/Nepal with some occurrences in Turkey [19], and *Datisca glomerata* restricted to California and Northern Mexico [20]. Members of the genus *Coriaria* (Coriariaceae, Cucurbitales) have the broadest geographic range. They can be found in New Zealand, Papua New Guinea, the Philippines, Taiwan, Japan, China, Nepal, Pakistan, northern India, the Mediterranean, and the west coast of South America [7, 21]. New Zealand is the center of diversity for *Coriaria,* with eight endemic species, while in any of the other regions only one or two different species can be found.

Interestingly, the biodiversity of *Frankia* cluster-2 strains in Eurasia is very low. There is 96–99.8% mean average nucleotide identity (ANI) between MAGs sequenced from nodules of inocula originating in France, Pakistan and Japan [14]. However, a MAG from an inoculum originating in Papua New Guinea (*Candidatus* Frankia meridionalis Cppng1) shows only ca. 85% mean ANI with the Eurasian *Frankia* cluster-2 MAGs [14]. Given that the symbiosis had evolved in Gondwana, it would seem plausible that *Frankia* cluster-2 split already into different lineages in Gondwana or the southern hemisphere. The question arises how did the *Frankia* cluster-2 and their host plants dispersed from Gondwana to Eurasia? We hypothesize that several lineages could have dispersed in the southern hemisphere, while only a few –or one– of them might have reached the northern hemisphere.

Therefore, in the hope to find representatives of a second *Frankia* cluster-2 lineage in the northern hemisphere, we obtained nodules from *Coriaria intermedia* from Taiwan and from the Philippines, as well as from *Coriaria japonica* in Japan, to analyse the corresponding *Frankia* cluster-2 MAGs.

## Results and discussion

### Sequencing of *Frankia* MAGs from *Coriaria intermedia* growing in Taiwan and the Philippines, and from *Coriaria japonica* growing in Japan

The *Frankia* metagenome-assembled genomes (MAGs, 17) obtained in this study were named according to the nomenclature established by Nguyen et al. [14]: [name of inoculum]_[initials of host plant from which DNA was isolated]_[“nod” for direct isolation of mixed plant and bacterial DNA from nodules, “vc” for isolation of DNA from vesicle clusters isolated from nodules].

**Fig. 1:**
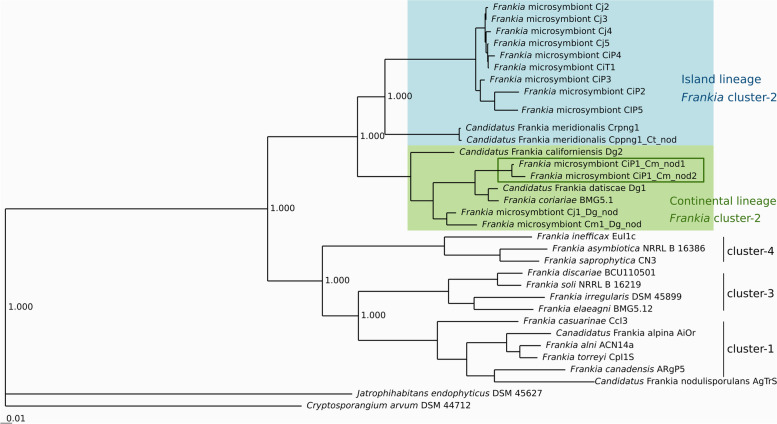
Phylogenetic tree based on whole-genome sequences inferred using the neighbor-joining algorithm as implemented in the PHYLIP package [22]. The tree was built for 34 *Frankia* genomes out of a core of 214 genes per genome, 7276 in total, by EDGAR 2.0 [23, 24]. The core has 92,816 amino acid residues per genome, 3,155,744 in total. Genomes used were *Candidatus* Frankia datisca Dg1 (NC_015656.1), *Frankia coriariae* BMG5.1 (NZ_JWIO00000000.1), *Candidatus* Frankia californiensis Dg2 (FLUV00000000.1), *Candidatus* Frankia meridionalis Cppng1 (CADDZT010000001-CADDZT010000101), *Frankia asymbiotica* M16386 (NZ_MOMC00000000.1), *Frankia saprophytica* CN3 (NZ_AGJN00000000.2), *Frankia inefficax* EuI1c (NC_014666.1), *Frankia irregularis* DSM45899 (NZ_FAOZ00000000.1), *Frankia elaeagni* BMG5.12 (NZ_ARFH00000000.1), *Frankia discariae* BCU110501 (NZ_ARDT00000000.1), *Frankia soli* NRRL B-16219 (MAXA00000000.1), *Frankia casuarinae* CcI3 (NC_007777.1), *Frankia canadensis* ARgP5 (GCF_900241035.1), *Candidatus* Frankia nodosporulans AgTrS (NZ_CADCWS000000000.1), *Candidatus* Frankia alpina AiOr (GCA_902806485), *Frankia alni* ACN14a (NC_008278.1), *Frankia torreyi* CpI1 (NZ_JYFN00000000.1) and *Frankia* sp. QA3 (AJWA00000000). The genomes of *Cryptosporangium arvum* DSM44712 (JFBT01000000) and *Jatrophihabitans endophyticus* DSM45617 (NZ_FQVU00000000.1) were added for rooting. Bootstrap values are 100 for every branch; they were calculated in R [25] using the packages APE [26] and phangorn [27]. The genomes sequenced from nodules of *Coriaria myrtifolia* induced by the inoculum from the Philippines, CiP1, are labeled by a green outline. The size bar denotes 0.01 changes

*Coriaria intermedia* nodules collected at Taiping Mountain (Taiwan) were used for DNA isolation, sequencing and the assembly of a *Frankia* cluster-2 MAG termed CiT1_Ci_nod (Tables 1 and 2). Six sets of *C. intermedia* nodules from Poblacion, Atok (Philippines) were available; the first set, CiP1, was used as inoculum. The five other nodule samples from Poblacion were used for DNA isolation and sequencing, yielding MAGs CiP2_Ci_nod to CiP6_Ci_nod (Tables 1 and 2). CiP2_Ci_nod consisted of two different strains too similar to each other to be separated by bioinformatics means; therefore, the MAG was not of high quality. CiP3_Ci_nod and CiP4_Ci_nod represented one strain each and showed good BUSCO values (91.9 and 87.2% BUSCO, respectively; Table 2; Supplementary Table S2). CiP5_Ci_nod and CiP6_Ci_nod were not of high quality since the metagenomes contained a large contribution of non*-Frankia* bacterial DNA. The inoculum CiP1 was used for cross-inoculation studies. DNA was isolated from nodules induced on the Mediterranean species *Coriaria myrtifolia* (CiP1_Cm_nod1, CiP1_Cm_nod2). These two *Frankia* MAGs were quite dissimilar from all other genomes going back to nodules from Poblacion (Table 2; Fig. 1).

**Table 1:** Inocula used and MAGs sequenced in this study. The Philippine inoculum CiP1 was used for cross-inoculation studies and was not sequenced on its original host

Host	Inocolum	Origin	Genome	Accession number
*Coriaria intermedia*	CiT1	Taiwain	CiT1_Ci_nod	CAKJTL010000001- CAKJTL010000192
*Coriaria intermedia*	CiP2	Philippines	CiP2_Ci_nod	CAKJTS010000001- CAKJTS010000335
*Coriaria intermedia*	CiP3	Philippines	CiP3_Ci_nod	CAKJTM010000001- CAKJTM010000241
*Coriaria intermedia*	CiP4	Philippines	CiP4_Ci_nod	CAKJTN010000001- CAKJTN010000207
*Coriaria intermeiia*	CiP5	Philippines	CiP5_Ci_nod	CAKJTT010000001- CAKJTT010001025
*Coriaria myrtifolia*	CiP1	Philippines	CiP1_Cm_nod	CAKKTM010000001- CAKKTM010000479
Coriaria myrtifolia	CiP1	Philippines	CiP1_Cm_nod2	CAKKTL010000001- CAKKTL010000399
*Coriaria japonica*	Cj2	Japan	Cj2_Cj_nod	CAKJTP010000001- CAKJTP010000304
*Coriaria japonica*	Cj2	Japan	Cj3_Cj_nod	CAKJTQ010000001- CAKJTQ010000325
*Coriaria japonica*	Cj4	Japan	Cj4_Cj_nod	CAKJTR010000001- CAKJTR010000337
*Coriaria japonica*	Cj5	Japan	Cj5_Cj_nod	CAKJTO010000001- CAKJTO010000213
*Coriaria terminalis*	Cppng1	Papua New Guinea	Cppng1_Ct_nod	CAKKTK010000001- CAKKTK010000151

**Table 2:** Features of MAGs sequenced in this study. The complete results of the BUSCO analysis are presented in Supplementary Table S2

Genome	Strains	Size (bp)	N50	Used Reads	Coverage	% GC	Completeness	***nod*** genes
CiT1_Ci_nod	1	4,987,545	37,416 bp	611,363	37x	67.04%	91.9% BUSCO	–
CiP2_Ci_nod	2	5,044,359	27,610 bp	511,456	30x	67.86%	48.8% BUSCO	*nodU*
CiP3_Ci_nod	1	6,243,892	8663 bp	412,578	19x	68.05%	91.9% BUSCO	*nodC-nltIJ- nodU*
CiP4_Ci_nod	1	5,207,054	42,806 bp	912,478	53x	67.93%	86.5% BUSCO	–
CiP5_Ci_nod	1	5,352,883	5918 bp	452,147	25x	67.80%	58.5% BUSCO	*nodC-nltIJ- nodU*
CiP1_Cm_nod1	1	5,200,215	16,642 bp	441,095	25x	71.03%	89.2% BUSCO	–
CiP1_Cm_nod2	1	5,375,179	20,496 bp	531,234	30x	71.04%	87.8% BUSCO	–
Cj2_Cj_nod	1	4,918,503	28,956 bp	520,408	32x	68.07%	91.2% BUSCO	–
Cj3_Cj_nod	1	5,373,832	24,916 bp	499,754	28x	67.92%	89.2% BUSCO	–
Cj4_Cj_nod	1	5,193,180	25,314 bp	498,419	29x	67.92%	84.5% BUSCO	–
Cj5_Cj_nod	1	5,233,754	41,892 bp	784,521	44x	67.99%	88.5% BUSCO	–
Cppng_Ct_nod	1	4,844,797	50,015 bp	658,720	41x	68.14%	91.2% BUSCO	*nodC-nltIJ- nodU*

DNA from four sets of nodules from *C. japonica* from Japan was isolated (Cj2_Cj_nod to Cj5_Cj_nod). The MAGs of these nodules were near-complete (84.5–91.2% BUSCO, Table 2). Unlike the Cj1_Dg_nod *Frankia* MAG which also originated from an inoculum from Japan [14], the MAGs of these strains did not contain any canonical *nod* genes which previously had been identified [12–14]. While neither the MAG CiT1_Ci_nod, CiP4_Ci_nod, CiP1_Cm_nod1 or CiP1_Cm_nod2 contained any representatives of the canonical *nod* genes, CiP3_Ci_nod and CiP5_Ci_nod contained a truncated version of the nod2 region identified in the genome of *Candidatus* Frankia meridionalis Cppng1, *nodC-nltIJ-nodU* (Supplementary Fig. S1 [14]). The *nod* gene status of CiP2_Ci_nod was unclear; it contained a *nodU* copy but not the rest of the operon, which might be related to the fact that it has only 48% BUSCO.

### The CiT1 and CiP1 inocula could nodulate *Coriaria* species from both hemispheres, but not *Datisca glomerata*

*Frankia* cluster-2 inocula usually have a broad host range, which may be related to the fact that they contain more than one strain [14]. While CiT1 could nodulate the New Zealand species *Coriaria arborea,* and CiP1 could nodulate the Mediterranean species *C. myrtifolia,* neither CiT1 nor CiP1 could nodulate *Datisca glomerata* (Table 3)*.* The *Frankia* MAGs of nodules induced by CiP1 on *C. myrtifolia* were sequenced (Tables 1 and 2) and shown to differ significantly from the MAGs isolated from the samples CiT1, CiP2-CiP5, and Cj2-Cj5 (Fig. 1).

**Table 3:** Host specificity analysis for different inocula. Data on Cppng1 and Dg1 were presented in Nguyen et al. [14]. PNG: Papua New Guinea. (nodulation)* - the MAG that was sequenced in the induced nodules belonged to a different lineage than the MAGs present in nodules induced on the original host

Inoculum			Taiwan	Philippines	PNG	Pakistan
			CiT1	CiP1	Cppng1	Dg1
Northern hemisphere	North America	***Datisca glomerata***	no nodulation	no nodulation	no nodulation	nodulation
	Eurasia	***Coriaria myrtifolia***	not tested	(nodulation)*	not tested	nodulation
		***Coriaria terminalis***	not tested	no nodulation	nodulation	nodulation
		***Coriaria intermedia***	nodulation	nodulation	not tested	no nodulation
Southern hemisphere	New Zealand	***Coriaria arborea***	nodulation	not tested	nodulation	no nodulation

### Cluster-2 *Frankia* strains from Papua New Guinea, the Philippines, Taiwan and Japan form a common lineage

To analyse the phylogeny of *Frankia* cluster-2, a core genome tree was constructed using the genomes of the type strains of all *Frankia* species available thus far as well as the MAGs obtained in this study. The results show that within *Frankia* cluster-2, two different lineages can be identified. On the one hand, *Candidatus* Frankia meridionalis Cppng1, the strains from Taiwan and the Philippines, and the novel strains from Japan form a common lineage. This lineage is distinct from the strains from the Eurasian continent represented by *Candidatus* Frankia datiscae Dg1 and *Frankia coriariae* BMG5.1, and the North American strains represented by *Candidatus* Frankia californiensis Dg2 (Fig. 1). Thus, we are calling the lineage represented by the MAGs Cppng1_Ca_nod, CiT1_Ci_nod, CiP2_Ci_nod to CiP5_Cj_nod, and Cj2_Cj_nod to Cj5_Cj_nod the ‘island lineage’ of *Frankia* cluster-2, in contrast with the ‘continental lineage’ represented by *Candidatus* F. datiscae, *F. coriariae*, *Candidatus* F. californiensis, and CiP1_Cm_nod1 and CiP1_Cm_nod2. Within the island lineage, the strains from the Philippines, Taiwan and Japan form a separate clade from *Candidatus* F. meridionalis.

CiP1_Cm_nod1 and CiP1_Cm_nod2, however, the *Frankia* MAGs from nodules induced by the CiP1 inoculum on the Mediterranean *Coriaria* species *C. myrtifolia,* clearly represented members of the continental lineage of *Frankia* cluster-2. Like members of *F. coriariae* [11, 16], they did not contain the canonical *nod* genes (Table 2). To ensure that no samples had been mixed up, we amplified and sequenced the plant *matK* phylogenetic markers from the raw data of the MAGs and confirmed host plant identity (Supplementary Table S3). In this context, we also wanted to confirm that the nodules induced by the inoculum from Papua New Guinea, Cppng1, on the Chinese *Coriaria* species *C. terminalis* [14] contained a MAG representing the island lineage. These nodules had been previously examined for *nod* gene expression using primers designed based on the Cppng1_Ca_nod sequence, indicating that indeed, the same strain was present in nodules of *C. arborea* and *C. terminalis* [14]. To settle any doubts, we sequenced the MAG from *C. terminalis* nodules (Cppng1_Ct_nod; Tables 1 and 2) which indeed showed 99.5% Average Nucleotide Identity (ANI) and 99.75% Average Amino Acid Identity (AAI) with Cppng1_Ca_nod (Supplementary Fig. S2; Supplementary Table S4A, B).

### Inocula from the Philippines and Japan can contain *Frankia* species from both the island lineage and the continental lineage

Average Nucleotide Identity (ANI) comparisons were performed for the 10 novel MAGs of cluster-2 included in the phylogenetic tree (Supplementary Fig. S2, Supplementary Table S4). Based on the usually applied ANI threshold range of 95–96% for species demarcation [28, 29], the mean ANI values presented in Supplementary Fig. S2 and Supplementary Table S4A show that the genomes from Japan, Taiwan and the Philippines represent a novel species. Because the mean ANI values for two MAGs with low BUSCO values, CiP2_Ci_nod and CiP5_Ci_nod, were below 95%, the analysis was repeated using AAI values (Supplementary Table S4B). Based on the results, all novel *Frankia* MAGs from Japan, Taiwan and the Philippines analysed in this study belong to the same species, different from *Candidatus* Frankia meridionalis. The only exceptions were the MAGs of CiP1_Cm_nod1 and CiP1_Cm_nod2, which belonged to the continental lineage (Fig. 1, Supplementary Fig. S2). They showed less than 90.1% ANI with those of *Frankia coriariae* BMG5.1 [30] or with *Candidatus* Frankia datiscae Dg1 [12] (Supplementary Fig. S2), indicating they form a novel species. Since nodulation of *C. terminalis* by the CiP1 inoculum failed (Table 3), these Eurasian lineage strains seemed to have a very narrow host specificity, or, more likely, they represented a very minor contribution to the CiP1 strain assemblage. Nodulation of *D. glomerata* by CiP1 also failed (Table 3), but this was expected since like *F. coriariae* BMG5.1, CiP1_Cm_nod1 and CiP1_Cm_nod2 do not contain the canonical *nod* genes and in our hands, no inoculum without the canonical *nod* genes could ever nodulate *D. glomerata* [14].

**Fig. 2:**
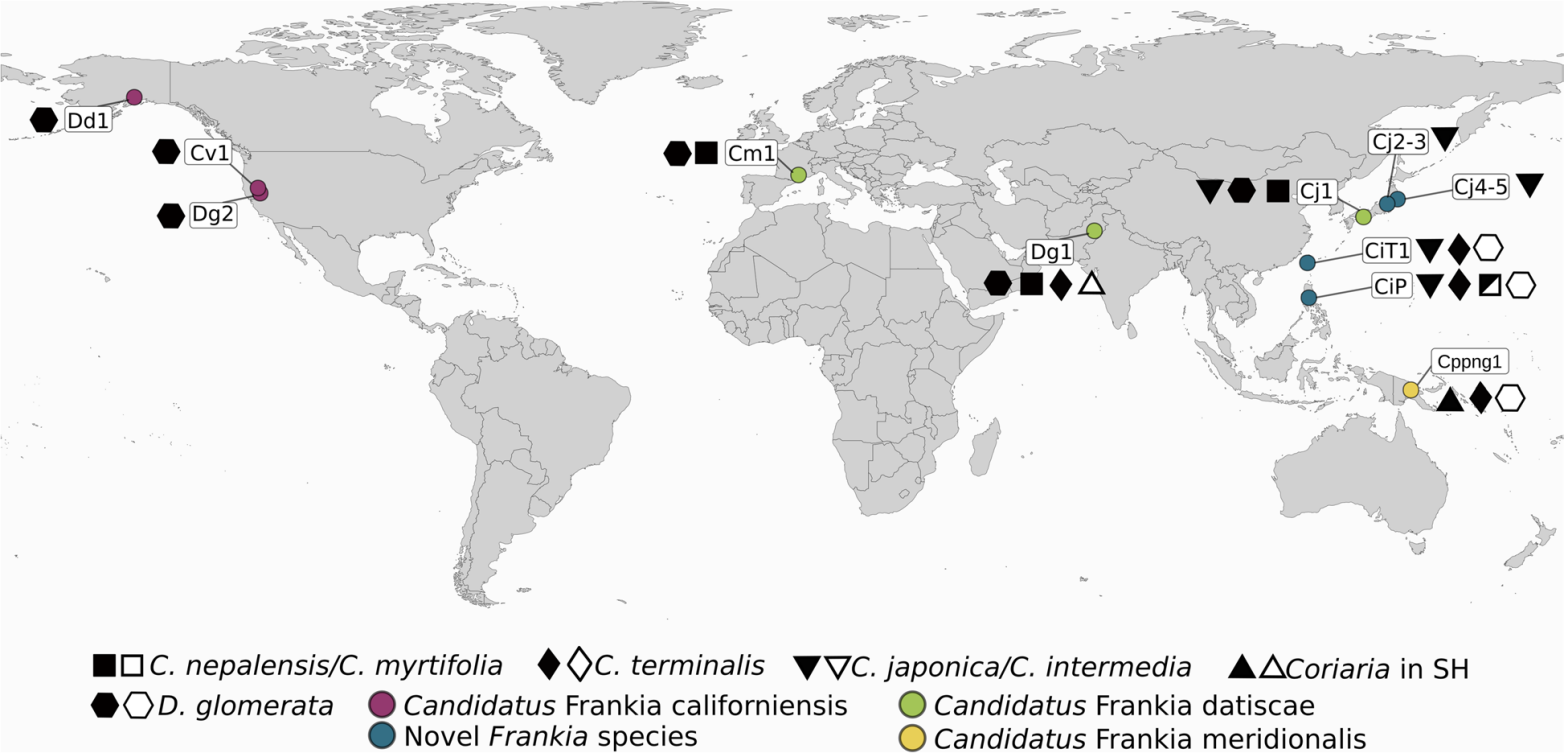
Map illustrating the location of *Frankia* samples and their host specificity. Black symbol: nodulation is possible. White symbol: nodulation was tried and was unsuccessful. Square: *Coriaria myrtifolia* or *Coriaria nepalensis;* diamond: *Coriaria terminalis;* down-facing triangle: *Coriaria japonica* or *Coriaria intermedia*; up-facing triangle: *Coriaria* sp. from the Southern hemisphere lineage; hexagon: *Datisca glomerata*. Colored dots of sampling represent species of *Frankia* as followed: magenta: *Candidatus* Frankia californiensis; green: *Candidatus* Frankia datiscae; blue: novel island lineage species presented in this study; yellow: *Candidatus* Frankia meridionalis, also part of the island lineage

Altogether, the strain assemblage that made up the inoculum CiP1 contained both representatives of a novel species of the island lineage and a novel species of the continental lineage. The island lineage was present in five different sets of *C. intermedia* nodules, while no sequences of a member of the continental lineage were obtained from nodules of this species (Supplementary Fig. S2; Fig. 2). It thus seems that while the strain assemblages contain both lineages, the members of the island lineage routinely outcompete the ones of the continental one when it comes to nodule induction on *C. intermedia*. It is likely that the members of the continental lineage are present on the outside of nodules. This has previously been shown for the cluster-3 species *Frankia irregularis* [31], members of which had been isolated from nodules of *Casuarina* species from different continents, but which cannot nodulate the *Casuarina* genus [32–34].

The *Frankia* MAGs from nodules of *D. glomerata* induced by an inoculum of *C. japonica* nodules (Cj1_Dg_vc and Cj1_Dg_nod) had been found to represent members of the species Ca. F. datiscae of the continental lineage; Cj1_Dg_nod also contained another genome that could not be separated bioinformatically [14]. Furthermore, the type strain of the species *F. coriariae,* BMG5.1, was isolated from nodules of *C. myrtifolia* which were induced by crushed nodules of *C. japonica* collected in Japan [11, 30]. Thus, inocula from *C. japonica* can contain strains of the continental lineage. Here we show that direct sequencing of *Frankia*-enriched metagenomes from *C. japonica* nodules yields representatives of the island lineage of *Frankia* cluster-2 (Table 2, Fig. 1, Fig. 2). In summary, *C. japonica* inocula contain strains of the continental as well as of the island lineage of *Frankia* cluster-2, but the latter outcompetes the former on *C. japonica*.

### What distinguishes the genomes of the island lineage from those of the continental lineage?

The MAGs of the island lineage all have a lower GC content than those of the continental lineage of *Frankia* cluster-2 (Table 2). A search for genes appearing in all MAGs available of members of the island lineage, but not in those of the continental lineage of cluster-2, revealed 230 genes (Supplementary Table S5A). The most interesting result was that all MAGs of the island lineage sequenced thus far contain the gene for 2-oxoglutarate dioxygenase (ethylene-forming; *efe*), first reported for *Pseudomonas syringae* strains [35, 36], later also for pathogenic fungi [37]. Ethylene production has been directly implicated in the pathogenicity of *P. syringae* pv. *glycinea,* though not of *P. syringae* pv. *phaseolicola* [38]. It is surprising that this enzyme is found in a plant symbiont. Based on Johansson et al. [37], the enzymes of strains of the island lineage of *Frankia* cluster-2 have all amino acid residues relevant for enzyme function (Supplementary Fig. S3). Upon investigation, we found that the *efe* gene was expressed in *Frankia,* in field nodules collected from *C. japonica* (Supplementary Fig. S4).

For legumes, it has been shown that ethylene inhibits symbiotic signalling [39] and also generally reduces nodulation via the root hair infection pathway [40]. This is confirmed by the fact that providing an enzyme that degrades the plant precursor of ethylene, 1-aminocyclopropane-1-carboxylate deaminase (AcdS), via the rhizobial microsymbionts themselves [41] or via rhizosphere bacteria [42], can improve nodulation. However, ethylene positively affects nodulation of peanut, which follows an intercellular infection pathway [43] and is required for nodulation of *Sesbania rostrata* via crack entry [44]. Thus, the negative effect of ethylene seems to be specific to nodulation via root hairs. Altogether, the presence and expression of *efe* genes in the MAGs of the strains of the island lineage suggest that nodulation by cluster-2 strains, at least in the case of *Coriaria* spp., requires ethylene and presumably follows an intercellular pathway.

A search for genes appearing in all MAGs available of members of the continental lineage, but not in MAGs of the island lineage of cluster-2, revealed 218 genes (Supplementary Table S5B). Only the MAGs from the continental lineage contain copies of the mammalian cell entry (*mce*) gene cluster from other actinobacteria like *Mycobacterium tuberculosis* (AEH09479 – AEH09484 in *Candidatus* Frankia datiscae Dg1) which in *Nocardia farcinia* was implicated in the interaction with, and invasion of, mammalian cells, and in *Streptomyces coelicolor* is required for plant root colonization [45, 46]. Furthermore, genes encoding the sodium/proton antiporter NhaA, which are present in all representatives of the continental lineage (AEH09573 and AEH09214 in *Candidatus* F. datiscae Dg1), were not found in the island lineage (Supplementary Table S5B), which could indicate lower levels of salt tolerance of the island lineage strains.

### How did *Frankia* cluster-2 strains spread from Gondwana across the world?

As mentioned above, the common ancestor of all root nodule-forming plants including the actinorhizal Cucurbitales evolved in Gondwana. Based on a fossil-dated phylogeny [7], a northern hemisphere clade of Coriariaceae (NH) diverged from the southern hemisphere clade of Coriariaceae (SH) in the Paleocene (ca. 57 Mya). The NH clade is split in *Coriaria nepalensis, Coriaria myrtifolia* and *Coriaria terminalis* on the one hand, and *Coriaria japonica* and *Coriaria intermedia* on the other hand. The SH clade of Coriariaceae encompasses the South American species *Coriaria ruscifolia*, the eight *Coriaria* species from New Zealand, and *Coriaria papuana* from Papua New Guinea. Why do the two parts of the NH clade of *Coriaria* have microsymbionts from different lineages of *Frankia* cluster-2, i.e. the continental or the island lineage?

The other family of actinorhizal Cucurbitales, Datiscaceae, evolved in India. This is based on a fossil wood of a precursor of the closely related Tetramelaceae, *Tetramelioxylon prenudiflora*, which has been found in the Deccan Intertrappean beds of Mohgaonkalan, Madhya Pradesh, India [47]. Thus, some Cucurbitales spread from Gondwana to Eurasia via India. The route via India would also be consistent with what is known about the phylogeny of the NH Coriariaceae [7].

Three scenarios are possible to explain how the two parts of the NH clade of *Coriaria* ended up with microsymbionts from different lineages of *Frankia* cluster-2. In the first one, *Datisca* dispersed to Eurasia via India together with its inoculum which represented the precursor of the continental lineage of *Frankia* cluster-2. The fact that *Frankia* cluster-2 strains from Papua-New Guinea, the Philippines, Taiwan and Japan belong to a common lineage, separate from the continental lineage, might suggest that *Frankia* cluster-2 spread from the SH to the NH via Papua New Guinea and Indonesia to the Philippines. The only extant host plant that fits this distribution would be *Coriaria*. *Coriaria* would then have dispersed to the mainland and would have eventually spread to areas where *Datisca* was growing. The strains of the continental lineage could have outcompeted the strains of the island lineage. This is unlikely based on the timing: given the changing distances between the islands involved, the proposed spread of *Coriaria* from the SH to the NH could have happened at the earliest near the end of the Oligocene 23 Mya. However, there is a *Coriaria* leaf fossil found in the Armissan bed in France which dates back to 23.2 to 33.9 Mya [7, 48]. Furthermore, this scenario does not account for the presence of the continental lineage of *Frankia* cluster-2 in Japan and the Philippines. In summary, this scenario can be excluded.

In the second scenario, *Datisca* and *Coriaria* came to Eurasia via India with *Coriaria* hosting both lineages of *Frankia* cluster-2. Eventually, the continental lineage outcompeted the island lineage because it was better adapted to the local soil or climate. However, the island lineage strains remained part of the strain assemblages. When *Coriaria* had spread from the mainland to the East Asian islands, the island lineage began to outcompete the continental lineage. This scenario is unlikely because it would have required uniform conditions on the Eurasian continent.

**Fig. 3:**
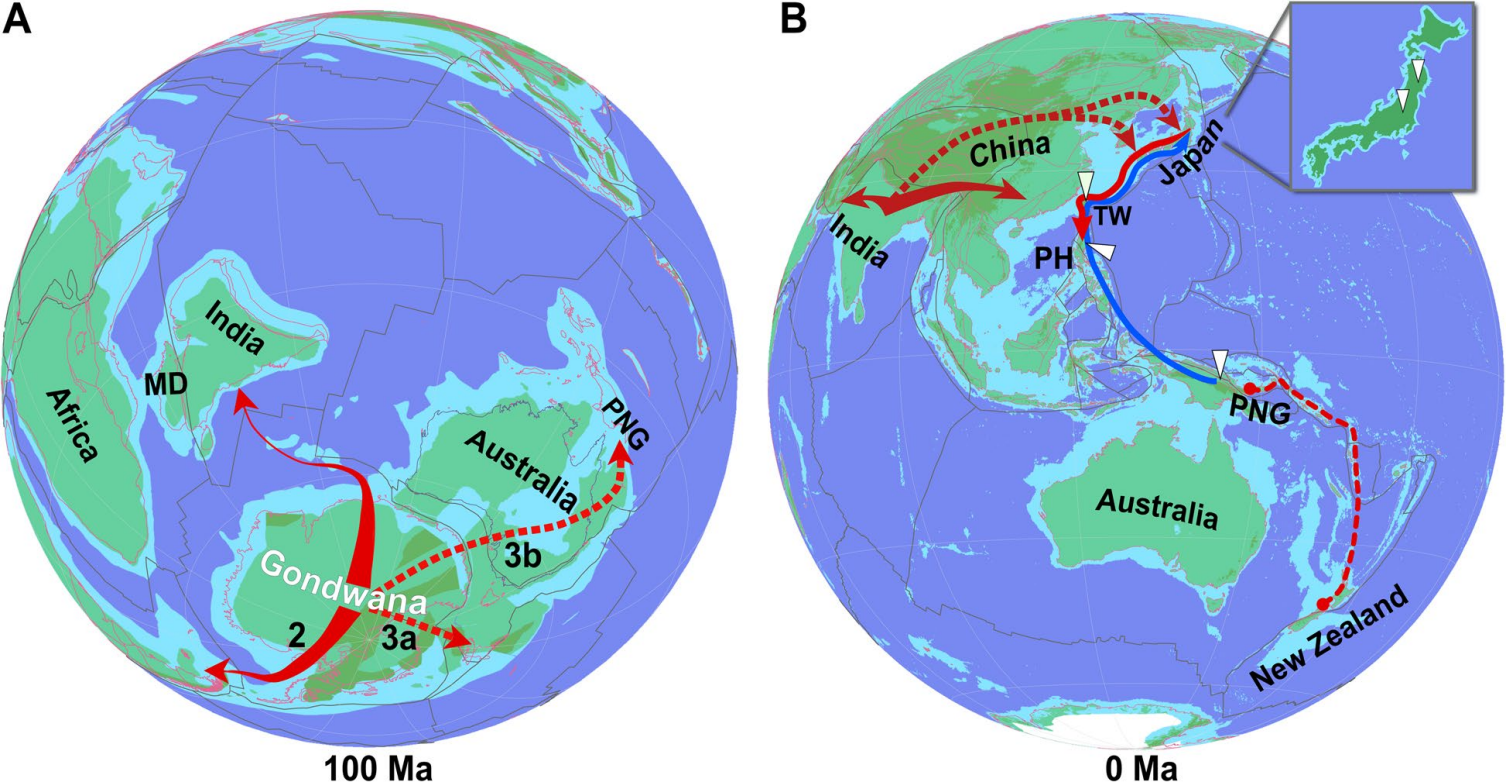
How cluster-2 *Frankia* strains spread from Gondwana across the world. (A) Geography from 100 mya, (B) current geography. Geography is according to the plate reconstruction of Zahirovic et al. [49] and paleo-environments from Cao et al. [50]. Land is given in green, deep sea in dark blue, and shallow sea as light blue. For reference, present-day coastlines and geological terrane boundaries are reconstructed using brown lines. Black lines indicate tectonic plate boundaries. Spread of *Coriaria* with cluster-2 *Frankia* is indicated by red arrows, hypothetical alternatives are indicated by dashed arrows. *Coriaria* nodule sampling points referred to in this study are indicated by white triangles. (A) *Coriaria* spread with the future continental cluster-2 lineage from Gondwana to India (1; continental lineage of *Frankia* cluster-2), and from Gondwana to South America (2; South American lineage). Distribution from Gondwana to New Zealand and Papua New Guinea (PNG; 3; island lineage of *Frankia* cluster-2) could have taken place via Australia (3a) or via New Zealand (3b) or in both directions simultaneously. (B) Distribution between PNG and New Zealand is ascertained by the fact that *Coriaria* is indigenous in all islands of the area [7], but the direction cannot be determined at this point (dashed line). When India had collided with Asia, *Coriaria* spread in Northern India-Pakistan-Nepal and from there westward to the Mediterranean and eastward into China. The precursor of *C. nepalensis* spread to the North (dashed arrow, since *Coriaria* is not found in Northern China today) towards Japan (*C. japonica*), and from there southward to Taiwan and the Philippines (*C. intermedia*); the separation of *C. japonica and C. intermedia* was recent (ca. 10 mya) [7]. The island lineage of *Frankia* cluster-2 spread from PNG to the Philippines and further to Taiwan (blue arrow), but the original host plants lost the symbiosis or went extinct. Strains of the island lineage outcompeted those of the continental lineage of *Frankia* when *Coriaria* spp. spread to Japan and from there to Taiwan and the Philippines. All cluster-2 *Frankia* strains sampled in continental Eurasia belong to the continental lineage. MD, Madagascar; PH, Philippines; PNG, Papua New Guinea; TW, Taiwan

In the third scenario, *Datisca* and *Coriaria* came to Eurasia via India, both with the continental lineage of *Frankia* cluster-2. In the Miocene, the island lineage spread northward from New Zealand or Papua New Guinea with another host plant. When *Coriaria* spread from the mainland to Japan/Taiwan/Philippines, the plants encountered the island lineage of *Frankia* cluster-2 which mostly outcompeted the continental lineage. Nevertheless, strains of the continental lineage remained part of some strain assemblages. The other host plant subsequently lost the symbiosis or became extinct. This is the most likely scenario and is depicted in Fig. 3.

How could the SH *Coriaria* clade and its microsymbiont come to Papua New Guinea? Based on the phylogeny of Renner et al. [7], *Coriaria* seems to have spread from Gondwana directly to New Zealand (see arrow 3a in Fig. 3A), and later, based on the position of *C. papuana* in the phylogeny, via Papua-New Guinea. Due to the disjunct distribution of the genus, the plant phylogeny cannot show how *Coriaria* spread from Gondwana to Papua-New Guinea; two interpretations are possible. Either *Coriaria* spread early to Papua-New Guinea via Australia where the genus would then have become extinct (see arrow 3b in Fig. 3A), or it spread from New Zealand to Papua-New Guinea, and later plants from Papua-New Guinea dispersed back to New Zealand.

## Conclusions

Strains of the earliest divergent *Frankia* clade, cluster-2, split into two lineages in Gondwana. One lineage came to the northern hemisphere via India with *Coriaria* sp. and the precursors of Datiscaceae. The other lineage spread in the southern hemisphere but eventually spread to the northern hemisphere when the distance between Papua New Guinea/New Guinea and the northern hemisphere South East Asian islands had reduced in the Miocene.

Loss of the symbiosis, or extinction of a symbiotic plant species, should have taken place for a non-*Coriaria Frankia* cluster-2 host after the split of the northern hemisphere *Coriaria* species in *C. myrtifolia, C. nepalensis, C. terminalis* on the one hand and *C. japonica, C. intermedia* on the other hand*,* i.e., at the earliest in the Miocene. This might be consistent with the hypothesis [8] that the decrease in atmospheric CO_2_ in the Oligocene and Miocene accounted for the loss of nodulation.

Analysis of inocula from *Coriaria* spp. nodules from New Zealand will have to show how cluster-2 *Frankia* strains reached this island.

## Methods

### Plant and bacterial material

Nodules of *Coriaria intermedia* Matsum. were collected at Taiping Mountain, Taiwan (24°29′51.5″ N 121°32′07.9″ E) in January 2017. Leaves were collected from the sample plant on July 8, 2018. A voucher was deposited in the herbarium of the Swedish Museum of Natural History, leg. K. Pawlowski s.n. (S; Reg. No. S18–40315). Nodules of *C. intermedia* were collected in April 2018 in Poblacion, Atok, Benguet/Sto. Tomas, Tuba, Benguet-CAR, Philippines. Vouchers were deposited in the Herbarium at the Department of Botany, University of the Philippines, leg. C.M. Bandong s.n. (Reg. No. 21414) and in the herbarium of the Swedish Museum of Natural History, leg. K. Pawlowski s.n. (S; Reg. No. S19–5452). Nodules of *Coriaria japonica* A. Gray were collected in RNAlater (Sigma-Aldrich, Japan) on the riverbanks of Nikko City (36°44′ N, 139°37′ E; 520 m a.s.l.) in central Japan by Sae Katayama and Masaki Tateno. Nodules were also collected in Oshu city (39°12′01.2“ N 141°23’48.1” E) in Iwate province in the northern part of Honshu, Japan, by Takashi Yamanaka in absolute ethanol. All nodules were immediately frozen and kept at − 20 °C upon arrival in Stockholm, Sweden. Vouchers were deposited in the herbarium of the Swedish Museum of Natural History, leg. F. Berckx s.n. (Reg. No. S22–86 and S22–87).

*Datisca glomerata* (C. Presl) Baill.*, Coriaria myrtifolia* L.*, Coriaria terminalis* Hemsl., *Coriaria arborea* Linds., and *C. intermedia* Matsum. were germinated from seeds in a greenhouse at 13 h light / 11 h dark, 25 °C in the light phase and 19 °C in the dark phase. *D. glomerata* seeds were obtained from plants in the greenhouse, going back to seeds collected in Vaca Hills, California. Seeds of *C. myrtifolia* were kindly provided by Paul Goetghebeur from the Botanical Garden at Gent University (Gent, Belgium). Seeds of *C. terminalis* var. *xanthocarpa* were purchased from www.plant-world-seeds.com. Seeds of *C. arborea* were kindly provided by Warwick Silvester. Seeds of *C. intermedia* were collected in Poblacion, Atok (Benguet-CAR, Philippines). After eight *(D. glomerata)* or twelve (*Coriaria* spp.) weeks, respectively, plantlets were inoculated with crushed nodules collected at Taiping Mountain (Taiwan) or in Poblacion, Atok (Philippines) and plants were maintained as described by Nguyen et al. [14]. Three to five months after inoculation, plants were inspected for nodulation status, and nodules were harvested into liquid nitrogen and kept at -80 °C.

### DNA isolation and sequencing

Total nodule DNA was isolated as described by Nguyen et al. [14] with small modifications. The nodules of *C. japonica* in the field were collected in RNAlater (Sigma-Aldrich, Japan) or ethanol, which was removed by gently washing with sterile milliQ water, followed by patting the nodules dry. DNA was isolated from *C. japonica* nodules using the NucleoSpin Plant II kit (Macherey-Nagel, Sweden). For isolation of DNA from nodules from *C. intermedia, C. terminals,* and *C. myrtfolia,* the GenElute™ Plant Genomic DNA Miniprep Kit (Sigma-Aldrich, Sweden) was used.

The genomic sequencing library of CiT1_Ci_nod was constructed from 1 ng of gDNA with the Nextera XT DNA Sample Preparation Kit (Illumina, Germany) according to the manufacturer’s protocol. The library was quality controlled by analysis on an Agilent 2000 Bioanalyzer with the Agilent High Sensitivity DNA Kit (Agilent Technologies, Germany) for fragment sizes of ca. 500–800 bp. Sequencing on a MiSeq sequencer (Illumina; 2 × 250 bp paired-end sequencing, v3 chemistry) was performed in the Genomics Service Unit (LMU Biocenter, Martinsried, Germany).

Libraries for genome sequencing of CiP2_Ci_nod, CiP3_Ci_nod, CiP4_Ci_nod, CiP5_Ci_nod, CiP6_Ci_nod, CiP1_Cm_nod, CiP1_Cm_nod2, Cj2_Cj_nod, Cj3_Cj_nod, Cj4_Cj_nod, Cj5_Cj_nod, and Cppng1_Ct_nod were constructed from 1 ng of DNA, sheared on Covaris M220 with Covaris MicroCaps 50 μl to approx. 600 bp, using the NEBNext Ultra II DNA Library Kit Kit (New England Biolabs, Germany) and the sparQ DNA Library Prep Kit (QuantaBio, MA, USA) following the manufacturer’s protocols. Libraries were quality controlled by analysis on an Agilent 2000 Bioanalyzer with the Agilent High Sensitivity DNA Kit (Agilent Technologies) for fragment sizes of ca. 500–800 bp. Sequencing was performed on a MiSeq sequencer as described above.

### Genome assembly and bioinformatics analyses

Assembly, binning and annotation were done as previously described [13, 14] with small modifications. In brief, de novo assembly was performed by applying the gsAssember 2.8. (Roche) with default settings. Raw reads were aligned to the corresponding assembled (meta-)genome contigs using Bowtie 2 (v2.4.1 [51];). The resulting SAM files were processed through SAMtools (v1.0 [52];). For binning, MetaBAT2 (v2.12.1 [53];) was applied with default settings. Raw reads were exported by mapping to the resulting *Frankia* bins and reassembled using again the gsAssembler 2.8 (Roche) with default settings. Completeness, contamination, and strain heterogeneity were estimated with BUSCO (v3.0.2 [54];), using the bacterial-specific single-copy marker genes database (odb9). For the annotation of the genomes, Prokka [55] and GenDB [56] were applied at default settings. Draft genome sequences were deposited at the EMBL/GenBank/DDBJ databases in BioProjects PRJEB47857 (CiT1), PRJEB47848 (CiP2), PRJEB47853 (CiP3), PRJEB47854 (CiP4), PRJEB47855 (CiP5), PRJEB47858 (Cj2). PRJEB47859 (Cj3), PRJEB47860 (Cj4), PRJEB47861 (Cj5), PRJEB48851 (CiP1_Cm_nod1), PRJEB48852 (CiP1_Cm_nod2). Core genome trees were inferred, average nucleotide identity was calculated, and genome comparisons were performed using the EDGAR 2.0 platform [23, 24]. Pairwise genome-to-genome distance calculations [digital DNA–DNA hybridization (dDDH)] were performed as recently described [57].

### Phylogenetic analysis of host plants

The phylogeny of the host plants was analysed based on the combination of the nuclear internal transcribed spacer (ITS) region, the large subunit of the ribulose-bisphosphate carboxylase gene (*rbcL*), maturase K (*matK*), or the chloroplast *trnL* gene. Primers were used as described in previous studies [7, 21]. All primers used in this study are listed in Supplementary Table S1.

### RNA isolation and quantitative reverse transcription polymerase chain reaction (RT-qPCR)

Total RNA was isolated from *C. japonica* nodules as described previously [14, 58] with some small modifications. In brief, nodules were washed in sterile MilliQ water to remove excess RNAlater (Sigma-Aldrich, Japan), and patted dry, and ground in liquid nitrogen with the addition of Polyclar AT. In the lysis buffer, samples were subjected to ultrasonication for three rounds at 30% pulsing, 25 s each round (ultrasonic homogenizer Sonoplus HD 2070, Bandelin Electronic, Berlin, Germany). RNA was extracted using the Spectrum Plant Total RNA kit from Sigma–Aldrich (Stockholm, Sweden) with on-column gDNA digestion (Sigma-Aldrich) according to the manufacturer’s instructions. Total removal of gDNA was verified using PCR with primers targeting the housekeeping gene *infC* [59], using cDNA of Cppng_Ca_nod as a positive control [14]. cDNA was then synthesized using TATAA GrandScript cDNA Synthesis Kit (TATAA, Sweden). Primers for the housekeeping gene *infC,* nitrogenase gene *nifD,* and 2-oxoglutarate dioxygenase (ethylene-forming) gene *efe* were designed using the primer design tool Primer3, available through NCBI. Primer efficiency between 90 and 110% was verified for each pair, and the best out of two designed pairs was chosen. Primer sequences are given in Supplementary Table S1. Each qPCR reaction contained 1x Maxima SYBR Green/ROX qPCR Master Mix (ThermoFisher Scientific), 300 nM of each primer, and 4 ng of cDNA in a reaction volume of 10 μl. The conditions of qPCR were as follows: 10 min at 95 °C, followed by 40 cycles of 15 s at 95 °C, 30 s at 60 °C, followed by a melt curve program of 15 s at 95 °C, 15 s at 60 °C, and 15 s at 95 °C. Gene expression values were normalized against *infC*, the gene encoding translation initiation factor IF3. The statistical analysis and data visualisation were performed in Rstudio [25].

## Supplementary Information

**Additional file 1.**

**Additional file 2.**

**Additional file 3.**

**Additional file 4.**

**Additional file 5.**

**Additional file 6.**

## Data Availability

Draft genome sequences were deposited at the EMBL/GenBank/DDBJ databases in BioProjects PRJEB47857 (CiT1), PRJEB47848 (CiP2), PRJEB47853 (CiP3), PRJEB47854 (CiP4), PRJEB47855 (CiP5), PRJEB47858 (Cj2), PRJEB47859 (Cj3), PRJEB47860 (Cj4), PRJEB47861 (Cj5), PRJEB48851 (CiP1_Cm_nod1), PRJEB48852 (CiP1_Cm_nod2).
