# Supplementary material for: A tale of two lineages: how the strains of the earliest divergent symbiotic Frankia clade spread over the world
Source: BMC Genomics. 2022 Aug 19;23:602. doi: 10.1186/s12864-022-08838-5 (PMC9392346; doi:10.1186/s12864-022-08838-5)
Supplement: Supplementary file 1 — Additional file 1. [file 12864_2022_8838_MOESM1_ESM.pdf]

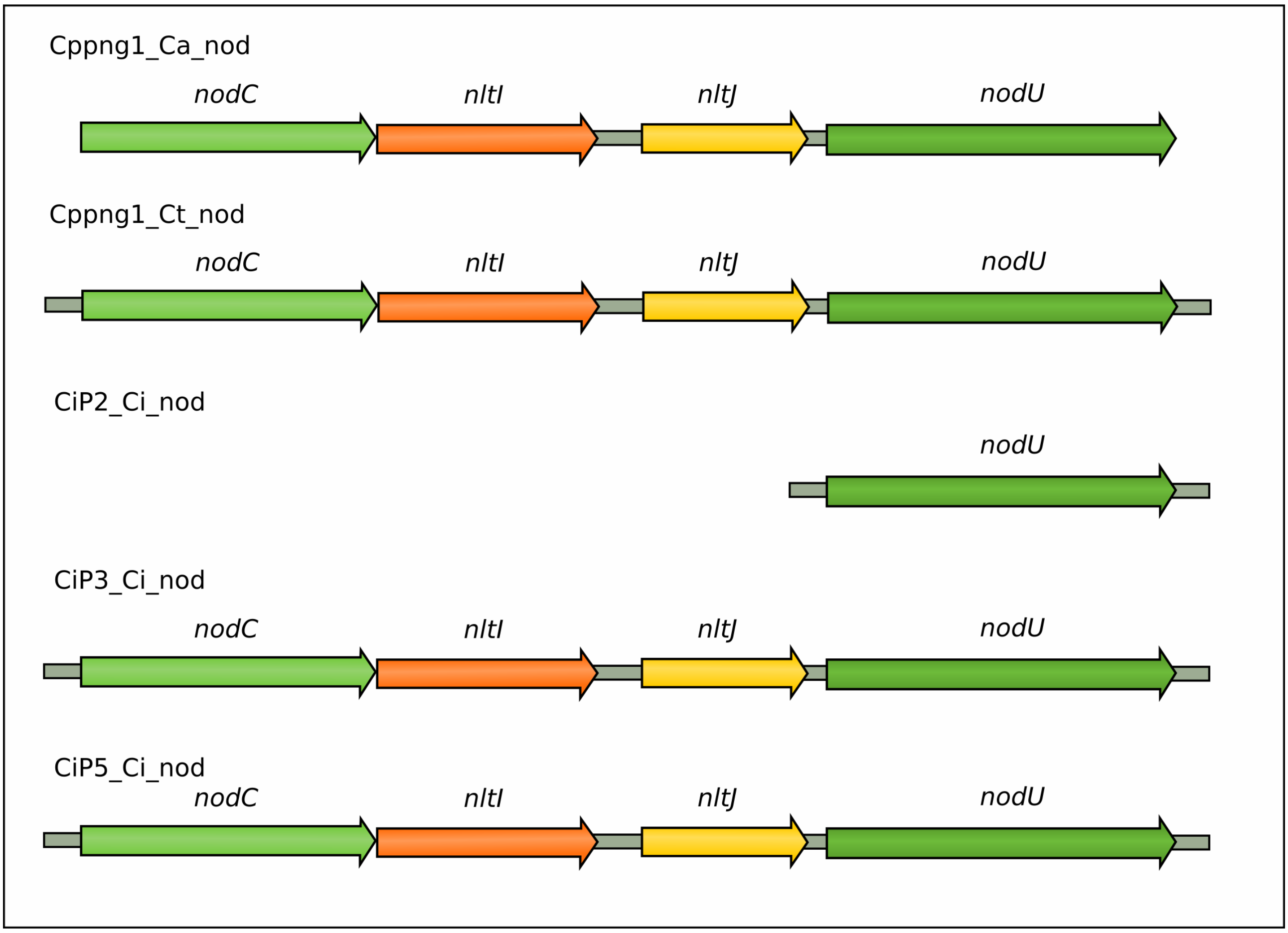

Supplementary Figure S1. comparison of the *nod* gene region of island lineage. Depicted are the *nodC* (light green), *niltI* (red), *nltJ* (yellow), and *nodU* (dark green) genes of the *nod* operon in *Candidatus* Frankia meridionalis Cppng1, and the three novel island genomes CiP3_Ci_nod and CiP5_Ci_nod. The genome of CiP2_Ci_nod was found to only contain the *nodU* gene. None of the other genomes of the island lineage in this study were found to contain *nod* genes.

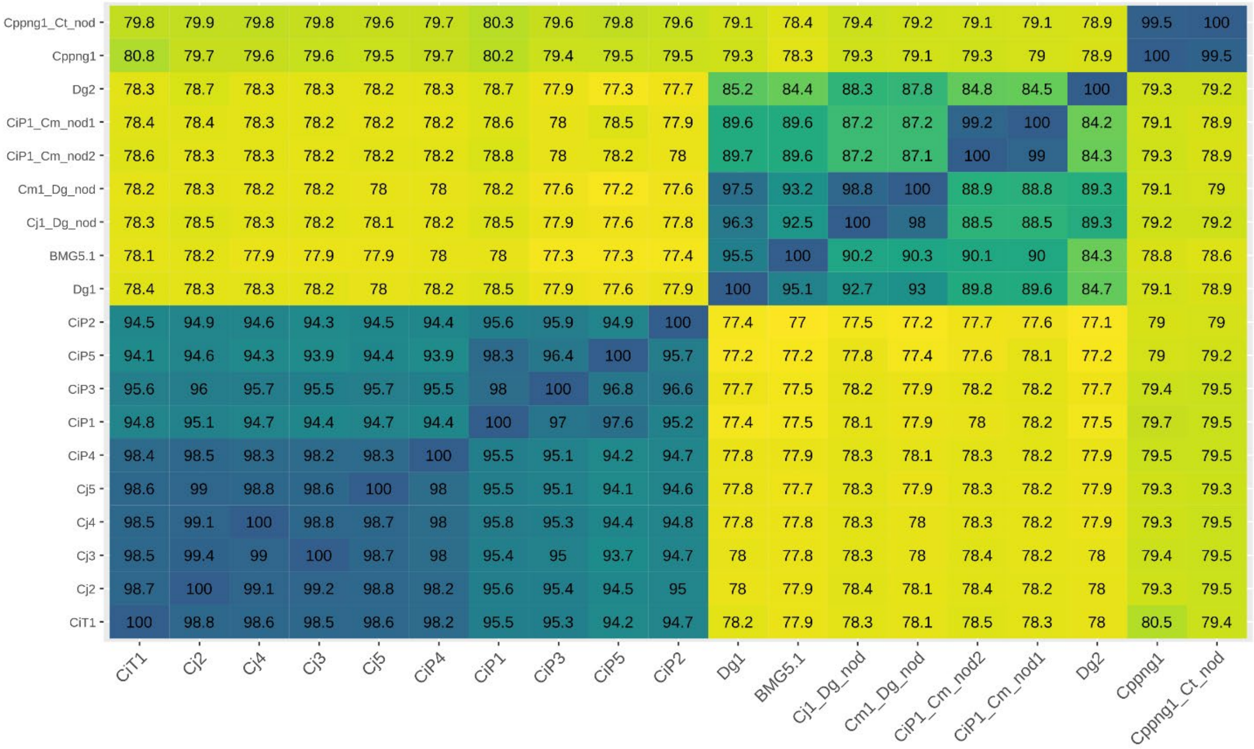

Supplementary Figure S2. Average Nucleotide Identity of cluster-2 genomes. Genomes included in analyses from top to bottom: *Candidatus* Frankia meridionalis Cppng1 (Cppng1_Ca_nod) and Cppng1_Ct_nod, followed by *Candidatus* Frankia californiensis Dg2, and a novel *Frankia* species represented by CiP1_Cm_nod1 and CiP1_Cm_nod2. These are followed by *Candidatus* Frankia datiscae Cj1 and Cm1, *Candidatus* Frankia datiscae Dg1, and *Frankia coriariae* BMG5.1. The genomes of CiT1, Cj2, Cj4, Cj3, Cj5, CiP4, CiP3, CiP5 and CiP2 represent a novel *Frankia* species of the island lineage. Heatmap depicts highest similarity in blue and lowest similarity in yellow.

```
                                          1
P.rubens_XP_002562422.1                   MLSHRSVSIA LRPPVICHNS IRHAPNLLAR QQTKG-LATT TAPSDLGSTM PPNYVARVGQ LKTFTLPEKA TGSPGDVEMG
P.savastanoi_pv.phaseolicola_AAD16440.1   ---------- ---------- ---------- ---------- ---------- -------MTN LQTFELPTEV TGCAADISLG
P.digitatum_EKV19239.1                    ---------- ---------- ---------- ---------- ---------M PPNYVARVGQ LKTFTLPETA TGSPSDVELG
CIP2_Ci_nod                               ---------- ---------- ---------- ------MTRS CYFGTRGSRT PAQKETELNE LQTFHLPEFI DGTESDLALA
CIP4_Ci_nod                               ---------- ---------- ---MPRV--- QPHPGTMTGS CYFGTRGSRT PTQKETELNE LQTFHLPEFI DGTESDRALA
CIP3_Ci_nod                               ---------- ---------- ---MPRV--- RPNPDAMTRS CYFGTRGSRT PAQKETELNE LQTFHLPEFI DGTESDLALA
CiT1_Ci_nod                               ---------- ---------- ---MPRV--- QPHPDTMTGS CYFGTRGSRT PTQKETELNE LQTFHLPEFI DGTESDRALA
Cj5_Cj_nod                                ---------- ---------- ---MPRV--- QPHPGTMTGS CYFGTRGSRT PTQKETELNE LQTFHLPEFI DGTESDRALA
Cppng1_Ct_nod                             ---------- ---------- ---------- ---------- ---------- -------MTD LQTFRLPEAV SGTESDIHLA
Cppng1_Ca_nod                             ---------- ---------- ---------- ---------- ---------- -------MTD LQTFRLPEAV SGTESDIHLA
Cj4_Cj_nod                                ---------- ---------- ---MPRV--- QPHPGTMTGS CYFGTRGSRT PTQKETELNE LQTFHLPEFI DGTESDRALA
Cj3_Cj_nod                                ---------- ---------- ---MPRV--- QPHPGTMTGS CYFGTRGSRT PTQKETELNE LQTFHLPEFI DGTESDRALA

                                          81
P.rubens_XP_002562422.1                   KALINAWRED GILQIAMNPK QQDLFNKAFA ASKRFFALPP NVKANCVDTQ SYAGYIASGE EITDGIADYS EIFTVTKDLP
P.savastanoi_pv.phaseolicola_AAD16440.1   RALIQAWQKD GIFQIKTDSE QDRKTQEAMA ASKQFCKEPL TFKSSCVSDL TYSGYVASGE EVTAGKPDFP EIFTVCKDLS
P.digitatum_EKV19239.1                    KAMINAWRED GILQVSMSPR QQALFENASA ASKRFFAMPP NQKAACVDTQ SYAGYIASGE EITDGIADYS EIFTVTKDLP
CIP2_Ci_nod                               RAMVRAWRSD GIFQVATSPA QDQKTQAAIE SSKRFFRLPM KAKSQCVSEL TYSGYIASGE EVTAGEADYS EIFTVCPDIA
CIP4_Ci_nod                               RAMVRAWRSD GIFQIATSPA QDQKTQAAIE SSKRFFRLPM KAKSQCVSEL TYSGYIASGE EVTAGEADYS EIFTVCPDIA
CIP3_Ci_nod                               RAMVRAWRSD GIFQIATSPA QDQKTQAAIE SSKRFFRLPM KAKSQCVSGL TYSGYIASGE EVTAGEADYS EIFTVCPDIA
CiT1_Ci_nod                               RAMVRAWRSD GIFQIATSPA QDQKTQAAIE SSKRFFRLPM KAKSQCVSEL TYSGYIASGE EVTAGEADYS EIFTVCPDIA
Cj5_Cj_nod                                RAMVRAWRSD GIFQIATSPA QDQKTQAAIE SSKRFFRLPM KAKSQCVSEL TYSGYIASGE EVTAGEADYS EIFTVCPDIA
Cppng1_Ct_nod                             REMIQAWRSD GIYQVATDPA QDRKTLEALE ASRRFFGMPM DFKARCISDL TYSGYIASGE EVTAGEADYS EIFTVCKDVP
Cppng1_Ca_nod                             REMIQAWRSD GIYQVATDPA QDRKTLEALE ASRRFFGMPM DFKARCISDL TYSGYIASGE EVTAGEADYS EIFTVCKDVP
Cj4_Cj_nod                                RAMVRAWRSD GIFQIATSPA QDQKTQAAIE SSKRFFRLPM KAKSQCVSEL TYSGYIASGE EVTAGEADYS EIFTVCPDIA
Cj3_Cj_nod                                RAMVRAWRSD GIFQIATSPA QDQKTQAAIE SSKRFFRLPM KAKSQCVSEL TYSGYIASGE EVTAGEADYS EIFTVCPDIA

                                         161
P.rubens_XP_002562422.1                   LEEPRVAAKW PCHGPCPWPD IDTKAPIQEY MDSLGSSGET LLQLIEHGLS LEP-KTLTSL TKDGWHHLRT LRFPQNNKTN
P.savastanoi_pv.phaseolicola_AAD16440.1   VGDQRVKAGW PCHGPVPWPN NTYQKSMKTF MEELGLAGER LLKLTALGFE LPI-NTFTDL TRDGWHHMRV LRFPPQTSTL
P.digitatum_EKV19239.1                    LDEPRVEAKW PCHGPCPWPD VDMRTPIQQY MDSLGKSGET LLQMIEYGLS LHP-DTLTSL TKDGWHHLRI LRFPQNNKTN
CIP2_Ci_nod                               LDDARVQARW PCHGPAPWPD AEYQRSMRVF MDELGRVGEK LLKLTALGLE LDDTDALTRL TADGWHHMRV LRFPALSKES
CIP4_Ci_nod                               LDDARVQARW PCHGPTPWPD AEYQRSMRVF MDELGRVGEK LLRLTALGLE LDDTDALTRL TADGWHHMRV LRFPALSKES
CIP3_Ci_nod                               LDDARVQARW PCHGPAPWPD AEYQRSMRVF MDELGRVGEK LLKLTALGLE LDDTDALTRL TADGWHHMRV LRFPALSKES
CiT1_Ci_nod                               LDDARVQARW PCHGPTPWPD AEYQRSMRVF MDELGRVGEK LLKLTALGLE LDDTDALTRL TADGWHHMRV LRFPALSKES
Cj5_Cj_nod                                LDDARVQARW PCHGPTPWPD AEYQRSMRVF MDELGRVGEK LLKLTALGLE LDDTDALTRL TADGWHHMRV LRFPALSKES
Cppng1_Ct_nod                             LDDARVQARW PGHGPAPWPD REYQQSMQVF MDELGSVGER LLKLTALGLE LDDTDALTKL AVDGWHHMRV LRFPARSEQT
Cppng1_Ca_nod                             LDDARVQARW PGHGPAPWPD REYQQSMQVF MDELGSVGER LLKLTALGLE LDDTDALTKL AVDGWHHMRV LRFPARSEQT
Cj4_Cj_nod                                LDDARVQARW PCHGPTPWPD AEYQRSMRVF MDELGRVGEK LLKLTALGLE LDDTDALTRL TADGWHHMRV LRFPALSKES
Cj3_Cj_nod                                LDDARVQARW PCHGPTPWPD AEYQRSMRVF MDELGRVGEK LLKLTALGLE LDDTDALTRL TADGWHHMRV LRFPALSKES

                                         241
P.rubens_XP_002562422.1                   GRGKEGRGIG SHTDYGLLVI AGQDEVGGLF IRPPYSDEKL -ENWK--SSA AGFREHDDRW TYVPPVPGVF TVFPGDMMQF
P.savastanoi_pv.phaseolicola_AAD16440.1   -----SRGIG AHTDYGLLVI AAQDDVGGLY IRPPVEGEKR NRNWLPGESS AGMFEHDEPW TFVTPTPGVW TVFPGDILQF
P.digitatum_EKV19239.1                    GRGKKGRGIG SHTDYGLLVI AAQDEVGGLF IRPPADDEKL -ENWK--NSA AGFREDDERW VYVPPVPGVF TVFPGDIMQF
CIP2_Ci_nod                               -----RRGIG AHTDYGLLVI AAQDDVGGLY IRPPVAGEKR NRNWLANESA AGMYENEEPW TFVRPVPSVL TVFPGDILQF
CIP4_Ci_nod                               -----RRGIG AHTDYGLLVI AAQDDVGGLY IRPPVAGEKR NRNWLANESA AGMYENEEPW TFVRPVPSVL TVFPGDILQF
CIP3_Ci_nod                               -----RRGIG AHTDYGLLVI AAQDDVGGLY IRPPVAGEKR NRNWLANESA AGMYENEEPW TFVRPVPSVL TVFPGDILQF
CiT1_Ci_nod                               -----RRGIG AHTDYGLLVI AAQDDVGGLY IRPPVAGEKR NRNWLANESA AGMYENEEPW TFVRPVPSVL TVFPGDILQF
Cj5_Cj_nod                                -----RRGIG AHTDYGLLVI AAQDDVGGLY IRPPVAGEKR NRNWLANESA AGMYENEEPW TFVRPVPSVL TVFPGDILQF
Cppng1_Ct_nod                             -----SRGIG AHTDYGMLVI AAQDDVGGLY IRPPVEGEQR NRNWLENESA AGMYENEQPW TFVKPVPSVL TVFPGDILQF
Cppng1_Ca_nod                             -----SRGIG AHTDYGMLVI AAQDDVGGLY IRPPVEGEQR NRNWLENESA AGMYENEQPW TFVKPVPSVL TVFPGDILQF
Cj4_Cj_nod                                -----RRGIG AHTDYGLLVI AAQDDVGGLY IRPPVAGEKR NRNWLANESA AGMYENEEPW TFVRPVPSVL TVFPGDILQF
Cj3_Cj_nod                                -----RRGIG AHTDYGLLVI AAQDDVGGLY IRPPVAGEKR NRNWLANESA AGMYENEEPW TFVRPVPSVL TVFPGDILQF

                                         321
P.rubens_XP_002562422.1                   MTNSYLPSTP HKVGLNTRER YAFAYFHEPS FQAEISPIAK LYDGKPPDEK NHYGTHFTNM FMRNYPDRVT TERILKEDRL
P.savastanoi_pv.phaseolicola_AAD16440.1   MTGGQLLSTP HKVKLNTRER FACAYFHEPN FEASAYPLFE ----PSANER IHYGEHFTNM FMRCYPDRIT TQRINKENRL
P.digitatum_EKV19239.1                    MTNSYLPSTP HKVGLNTRER FAFAYFHEPS FQAVVSPVAK LYDGQPPVEK IHYGTHFTNM FMRNYPDRIT TERIIKEDRL
CIP2_Ci_nod                               LTDGFLLSTP HKVRLNTRER FALAYFHEPN FAARVRPLSG ----N-SADC IHYGTHFTNM FMRSYPARIT TQRILAEDRL
CIP4_Ci_nod                               LTDSFLLSTP HKVRLNTRER FALAYFHEPN FAARIRPLSG ----V-SADC IHYGTHFTNM FMRSYPARIT TQRILAEDRL
CIP3_Ci_nod                               LTDGFLLSTP HKVRLNTRER FALAYFHEPN FAARVRPLSG ----T-SADC IHYGTHFTNM FMRSYPARIT TQRILAEDRL
CiT1_Ci_nod                               LTDSFLLSTP HKVRLNTRER FALAYFHEPN FAARIRPLSG ----V-SADC IHYGTHFTNM FMRSYPARIT TQRILAEDRL
Cj5_Cj_nod                                LTDDFLLSTP HKVRLNTRER FALAYFHEPN FAARIRPLSG ----V-SADC IHYGTHFTNM FMRSYPARIT TQRILAEDRL
Cppng1_Ct_nod                             LTNGFLLSTP HKVRLNTRER FALAYFHEPN FEAYLRPLSG ----L-GTEY IRYGTHFTNM FMRSYPERIT TRRILAEDRL
Cppng1_Ca_nod                             LTNGFLLSTP HKVRLNTRER FALAYFHEPN FEAYLRPLSG ----L-GTEY IRYGTHFTNM FMRSYPERIT TRRILAEDRL
Cj4_Cj_nod                                LTDGFLLSTP HKVRLNTRER FALAYFHEPN FAARIRPLSG ----V-SADC IHYGTHFTNM FMRSYPARIT TQRILAEDRL
Cj3_Cj_nod                                LTDGFLLSTP HKVRLNTRER FALAYFHEPN FAARIRPLSG ----V-SADC IHYGTHFTNM FMRSYPARIT TQRILAEDRL

                                         401
P.rubens_XP_002562422.1                   KLLDLPELRT K------
P.savastanoi_pv.phaseolicola_AAD16440.1   AHLEDLKKYS DTRATGS
P.digitatum_EKV19239.1                    QLLDRPELRT Q------
CIP2_Ci_nod                               SVLSLLRDDG GLLIS--
CIP4_Ci_nod                               SVL------- -------
CIP3_Ci_nod                               SVLSLLRDDG GLLIS--
CiT1_Ci_nod                               SVLPLLRDDG GLLIS--
Cj5_Cj_nod                                SVLPLLRDDG GLLIS--
Cppng1_Ct_nod                             SFFVAAR--- -------
Cppng1_Ca_nod                             SFFVAAR--- -------
Cj4_Cj_nod                                SVLPLLRDDG GLLIS--
Cj3_Cj_nod                                SVLPLLRDDG GLLIS--
```

Supplementary Figure S3: amino acid sequence alignment of the *efe* gene. Sequences were taken from *Penicillium rubens*, *Pseudomonas savastanoi pv. phaseolicola, Penicillium digitatum,* CiP2_Ci_nod, CiP4_Ci_nod, CiP3_Ci_nod, CiT1_Ci_nod, Cj4_Ci_nod, Cppng1_Ct_nod, Cppng1_Ca_nod, Cj4_Cj_nod, and Cj3_Cj_nod. Sequences were aligned using Clustal O, implemented through SeaView.

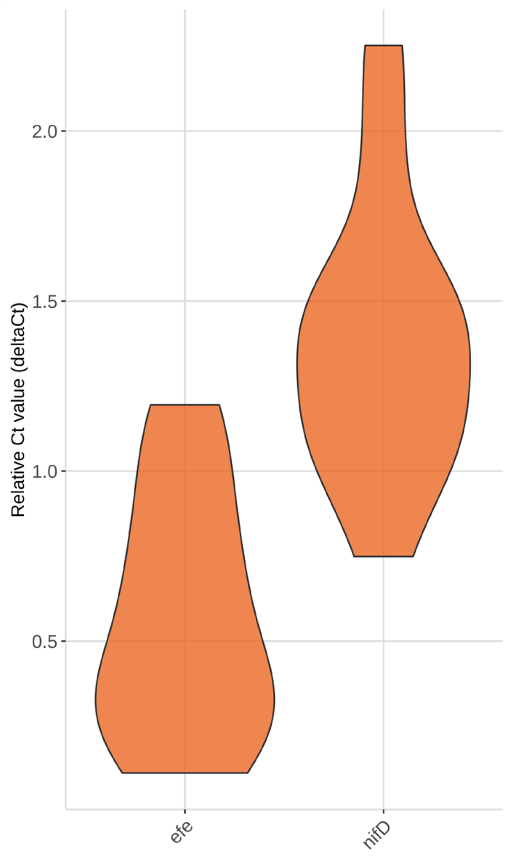

Supplementary Figure S4: relative *efe* gene expression levels compared to the structural nitrogenase gene *nifD.* Gene expression was analysed in nodules of *Coriaria japonica,* from the same plant which led to the genomes of Cj2_Cj_nod and Cj3_Cj_nod. Relative expression was normalized against the housekeeping gene *infC* encoding the translation initiation factor IF-3 (Alloisio et al. 2010).

Supplementary Figure references:

Alloisio N, Queiroux C, Fournier P, Pujic P, Normand P, Vallenet D, Médigue C, Yamaura M, Kakoi K, Kucho KI. (2010) The *Frankia alni* symbiotic transcriptome. *Molecular Plant-Microbe Interactions* **23:** 593–607.

Johansson N, Persson KO, Larsson C, Norbeck J (2014) Comparative sequence analysis and mutagenesis of ethylene forming enzyme (EFE) 2-oxoglutarate/Fe(II)-dependent dioxygenase homologs. *BMC Biochemisty* **15:** 22.
